# Identification of echinoderm metabolites as potential inhibitors targeting wild-type and mutant forms of Escherichia coli RNA polymerase (RpoB) for tuberculosis treatment

**DOI:** 10.1371/journal.pone.0304587

**Published:** 2024-08-30

**Authors:** Fatimah M. Alsulais, Bayan A. Alhaidhal, Ramzi A. Mothana, Abdullah R. Alanzi

**Affiliations:** Department of Pharmacognosy, College of Pharmacy, King Saud University, Riyadh, Saudi Arabia; Ahram Canadian University, EGYPT

## Abstract

Tuberculosis (TB) remains a critical global health challenge, with the emergence of drug-resistant strains heightening concerns. The development of effective drugs targeting both wild-type (WT) and mutant *Escherichia coli* RNA polymerase β subunit (RpoB) is crucial for global TB control, aiming to alleviate TB incidence, mortality, and transmission. This study employs molecular docking and ADMET analyses to screen echinoderm metabolites for their potential inhibition of *Escherichia coli* RNA polymerase, focusing on wild-type and mutant RpoB variants associated with TB drug resistance. The evaluation of docking results using the glide gscore led to the selection of the top 10 compounds for each protein receptor. Notably, CMNPD2176 demonstrated the highest binding affinity against wild-type RpoB, CMNPD13873 against RpoB D516V mutant, CMNPD2177 against RpoB H526Y mutant, and CMNPD11620 against RpoB S531L mutant. ADMET screening confirmed the therapeutic potential of these selected compounds. Additionally, MM-GBSA binding free energy calculations and molecular dynamics simulations provided further support for the docking investigations. While the results suggest these compounds could be viable for tuberculosis treatment, it is crucial to note that further in-vitro research is essential for the transition from prospective inhibitors to clinical drugs.

## 1. Introduction

Tuberculosis (TB) is an infectious bacterial infection caused mostly by *Mycobacterium tuberculosis* (Mtb). It primarily affects the lungs, although other regions of the body may be affected as well. TB remains a global health threat, necessitating ongoing research into novel therapeutic strategies to combat this infectious disease. Targeting bacterial RNA polymerase (RNAP) is a promising approach due to its role in gene transcription and the importance of the β subunit (RpoB) in bacterial viability. *Escherichia coli*, a model organism for studying bacterial transcription, contains an RNA polymerase that is structurally and functionally similar to that of Mtb [1–3]. Hence, studying inhibitors that can target both wild-type and mutant forms of *E*. *coli* RNA polymerase provides important insights for developing broad-spectrum antibacterial agents against TB.

RpoB, the β subunit of bacterial RNA polymerase, is critical for transcription initiation and elongation. Mutations in the RpoB gene, particularly in the rifampicin resistance-determining region (RRDR), cause resistance to rifampicin, a first-line antibiotic in tuberculosis treatment. Addressing the issue of rifampicin-resistant strains is critical for the development of new therapies, making RpoB an appealing target for drug discovery [4–7].

The search for new tuberculosis treatments has led researchers to look for bioactive compounds with antimycobacterial properties in nature. Several natural metabolites have been studied for their potential role in tuberculosis (TB) treatment [8]. Among these, plant-derived compounds have received a lot of attention. Berberine, an alkaloid isolated from plants such as Berberis vulgaris, has been shown to have antimycobacterial activity by disrupting bacterial cell membrane integrity and inhibiting mycobacterial DNA replication [9]. Furthermore, flavonoids like quercetin, which can be found in a variety of fruits and vegetables, have antimycobacterial properties by disrupting the integrity of mycobacterial cell walls and modulating host immune responses [10]. Furthermore, polyphenols derived from the rhizomes of Curcuma longa (turmeric) have been shown to have antimycobacterial activity through a variety of mechanisms, including virulence factor suppression and mycobacterial growth inhibition [11]. These natural metabolites provide promising leads for the development of novel tuberculosis therapies, either as standalone treatments or as adjuncts to existing antibiotic regimens, highlighting the potential of natural products in combating this global infectious disease.

Notably, marine organisms such as echinoderms have received attention for their diverse array of bioactive metabolites with pharmacological applications. Echinoderm metabolites can be classified as steroids, glycosides, ceramide derivatives, and other substances [12, 13] Echinoderm metabolites have been studied for a variety of other biomedical applications with various pharmacological activities, such as anticancer, anti-inflammatory, antiviral, and antioxidant properties [14]. For example, glycosaminoglycans (GAGs) extracted from sea cucumbers have demonstrated potential in wound healing and tissue regeneration due to their ability to modulate cellular processes involved in tissue repair [15, 16]. Furthermore, echinoderm-derived metabolites have been studied for their neuroprotective properties, with compounds like cerebrosides and sulfated polysaccharides showing promise in the treatment of neurodegenerative diseases [17]. These findings highlight the adaptability of echinoderm metabolites and their importance in drug discovery and development across multiple therapeutic areas [18]. Previous research has identified echinoderms as promising candidates for tuberculosis treatment. For example, holothurins, a type of triterpene glycoside found in sea cucumbers, have shown antimycobacterial activity against Mtb [19, 20]. Additionally, saponins extracted from sea cucumbers have shown inhibitory effects against drug-resistant Mtb strains, highlighting the therapeutic potential of marine-derived compounds in combating TB [21, 22].

This study aims to use echinoderm metabolites, which have shown promise in a variety of biological activities, to inhibit E. coli RNA polymerase. These metabolites may provide a dual mechanism of action by targeting both wild-type and mutant forms of RpoB, resulting in a comprehensive strategy against tuberculosis, including drug-resistant strains.

## 2. Methodology

### 2.1. Ligand preparation

The library of echinoderm metabolites containing 1600 compounds was obtained from the Comprehensive Marine Natural Products Database (https://www.cmnpd.org/). The ligand structures were prepared for further study using the LigPrep program from Schrödinger’s Maestro software package [23]. For every ligand, conformers were produced, and geometries were optimized using LigPrep. For each ligand in this instance, 32 conformers were constructed to account for their different orientations and potential flexibility. The OPLS_2005 forcefield was used to adjust the ligands’ geometry to guarantee that they were in conformations that were energetically favorable. The OPLS_2005 forcefield is a common empirical forcefield for calculating and showing the energy of interactions between atoms in molecules [24]. By reducing the energy of the compounds, any unfavorable interactions or strained geometry were removed.

### 2.2. Protein preparation

The goal of this study is to elucidate the virtual screening of the echinoderm’s metabolites against Escherichia coli RNA polymerase and its mutants to find the most suitable hits that can be pushed towards the wet lab studies. The proteins used in this study were Escherichia coli RNA polymerase wildtype (PDB ID: 5UAC), RpoB D516V mutant (PDB ID: 5UAH), RpoB H526Y mutant (PDB ID: 5UAQ), and RpoB S531L mutant (PDB ID: 5UAL) were retrieved from PDB and prepared for docking by Protein Preparation Wizard, included in the Schrödinger Maestro software suite [25]. There were several processes involved in the preparation of protein. Bond orders were set, disulfide bonds were created, and zero-order metal bonds were allocated. Additionally, hydrogen was added to the protein structures. All additional water molecules and ligands were eliminated from the crystal structures. Using the PROPKA program, we calculated the protein ionizable groups’ pKa values [26], and proteins’ hydrogen bond networks were optimized at pH 7.0. Finally, OPLS_2005 forcefield was used to minimize protein energy. After the protein was prepared, 3D grids were built at the reported sites of the respective proteins. Using these grids, to examine the interactions and binding patterns, ligands were docked into pre-specified sites of binding on proteins. This process is known as site-specific docking.

### 2.3. Molecular docking

The Glide docking module was used in SP (Standard Precision) mode to dock the prepared ligands at specific sites on the prepared protein structures [27]. The gliding scores of the docked ligands were analyzed and selected.

### 2.4. ADMET analysis

To determine their ADMET (absorption, distribution, metabolism, excretion, and toxicity) and physicochemical properties, the docked ligands underwent a comprehensive analysis. To achieve this, Maestro’s QikProp tool was employed, providing predictions for various attributes based on the ligands’ molecular structures [28]. Molecular weight, hydrogen bond acceptors, Hydrogen bond donors, QPlogBB, QPPCaco, QPlogKhsa, QPlogPo/w, and QPlogHERG were important characteristics. Hydrogen bond donors and acceptors are metrics that quantify the number of atom centers and hydrogen atoms available for participating in interactions involving hydrogen bonds. The logarithm of the octanol and water partition coefficients is predicted by QPlogPo/w, which provides information about the compound’s membrane permeability and hydrophobicity. QPlogHERG assesses the potential of a ligand to block the hERG potassium channel, providing information about the likelihood of cardiac toxicity. QPPCaco is a model for intestinal absorption that determines a compound’s permeability over the monolayer of Caco-2 cells. The substance’s ability to penetrate the blood-brain barrier and reach the central nervous system is indicated by QPlogBB, which forecasts the BBB partition coefficient’s logarithm. Finally, the logarithm of the binding affinity to human serum albumin, a necessary protein that influences drug distribution and binding efficiency, is determined by QPlogKhsa.

### 2.5. MD simulation

Desmond was employed to conduct molecular dynamics simulations lasting 100 ns for selected compounds [29]. We performed molecular dynamics simulations to evaluate the stability of the protein and ligand complexes. Molecular Dynamics simulation was used to evaluate the stability of complexes after several stages, including preprocessing, optimization, and reduction. Minimization was done using the OPLS_2005 force field [24]. The complexes were solvated in a periodic box with a 10 Å size containing the TIP3P water molecules [30]. Neutralization of the systems was done by adding counter ions and 0.15 M NaCl salt as needed to mimic physiological conditions. A pressure of 1 atm and a temperature of 300 K were set using the NPT ensemble. The systems went through a relaxing period before the simulation started. Trajectories were recorded and saved at 40 ps intervals during the simulation, allowing for a later study of the outcomes.

## 3. Results

### 3.1. Structural comparison

**Fig 1:**
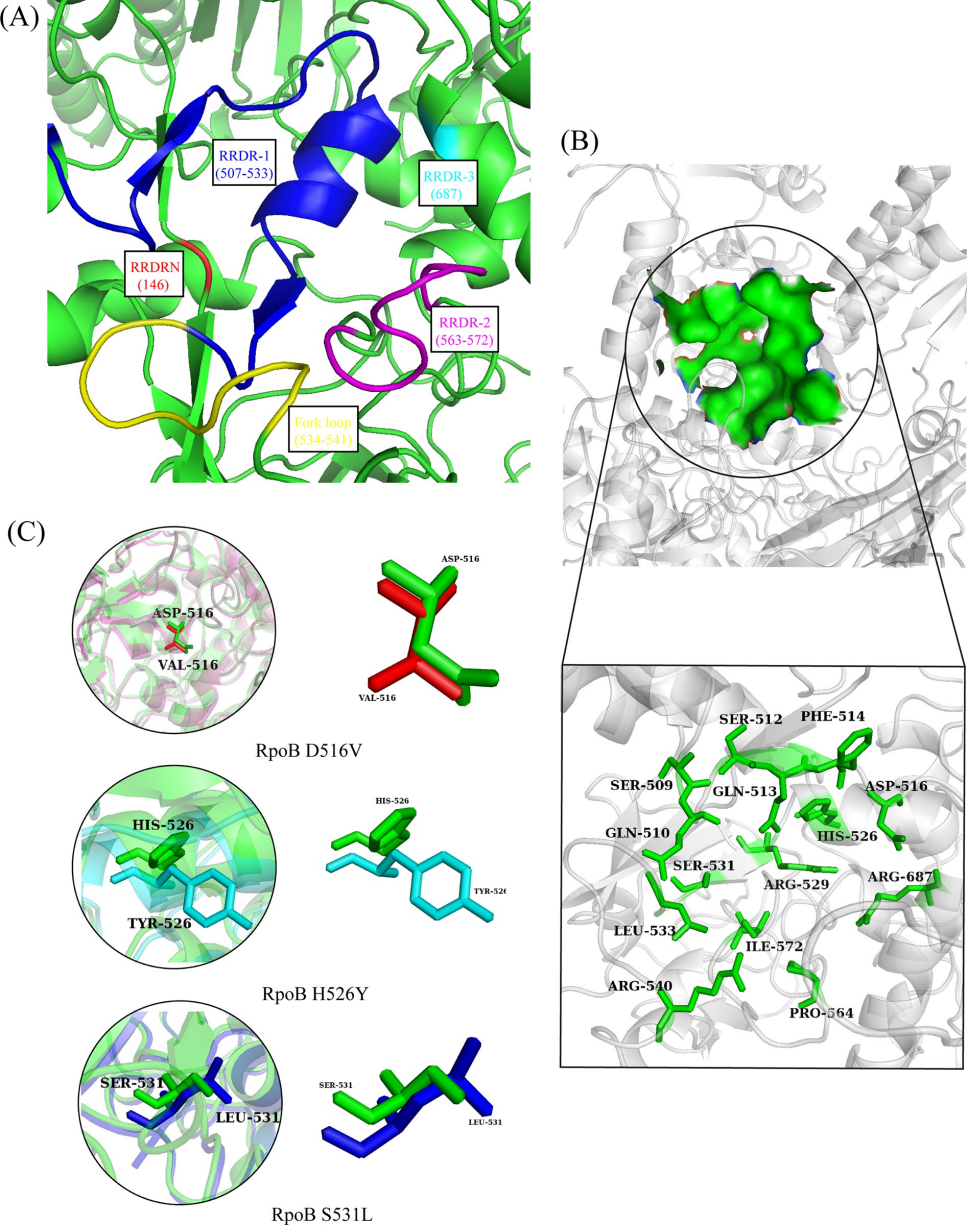
(A) The representation of resistance determining regions (RRDRs) in the RpoB structure. The four regions are represented by different colors, additionally, fork loop is also represented by yellow color. (B) The representation of the protein binding pocket in surface shape and labeled residues. (C) The comparison of mutated structures with wildtype, indicating the presence of mutations in the binding pocket.

The Rifamycin and its synthetic derivates have been used against mycobacterial infections for a long time with promising effects till 1960. The antibiotic Rifamycin targets the bacterial RNA polymerase to block the extension of RNA beyond the length of 2–3 nt. The binding site of the RNA polymerase is located on the beta subunit (RpoB). With time, the bacterial strains developed resistance against the antibiotics due to the mutations in the RpoB structure. The mutations were reported in the four regions including N-terminal cluster (residue 146), cluster 1 (507–533), cluster 2 (563–572), and cluster3 (687) (Fig 1A) [31]. The reported mutations in the RpoB structures were mainly single amino acid substitution observed in the cluster 1, highly conserved region in the bacterial RNA polymerases. Further, it was observed that all the mutational sites were in the binding site of the protein (Fig 1B and 1C).

### 3.2. Molecular docking and AMDET analysis

**Fig 2:**
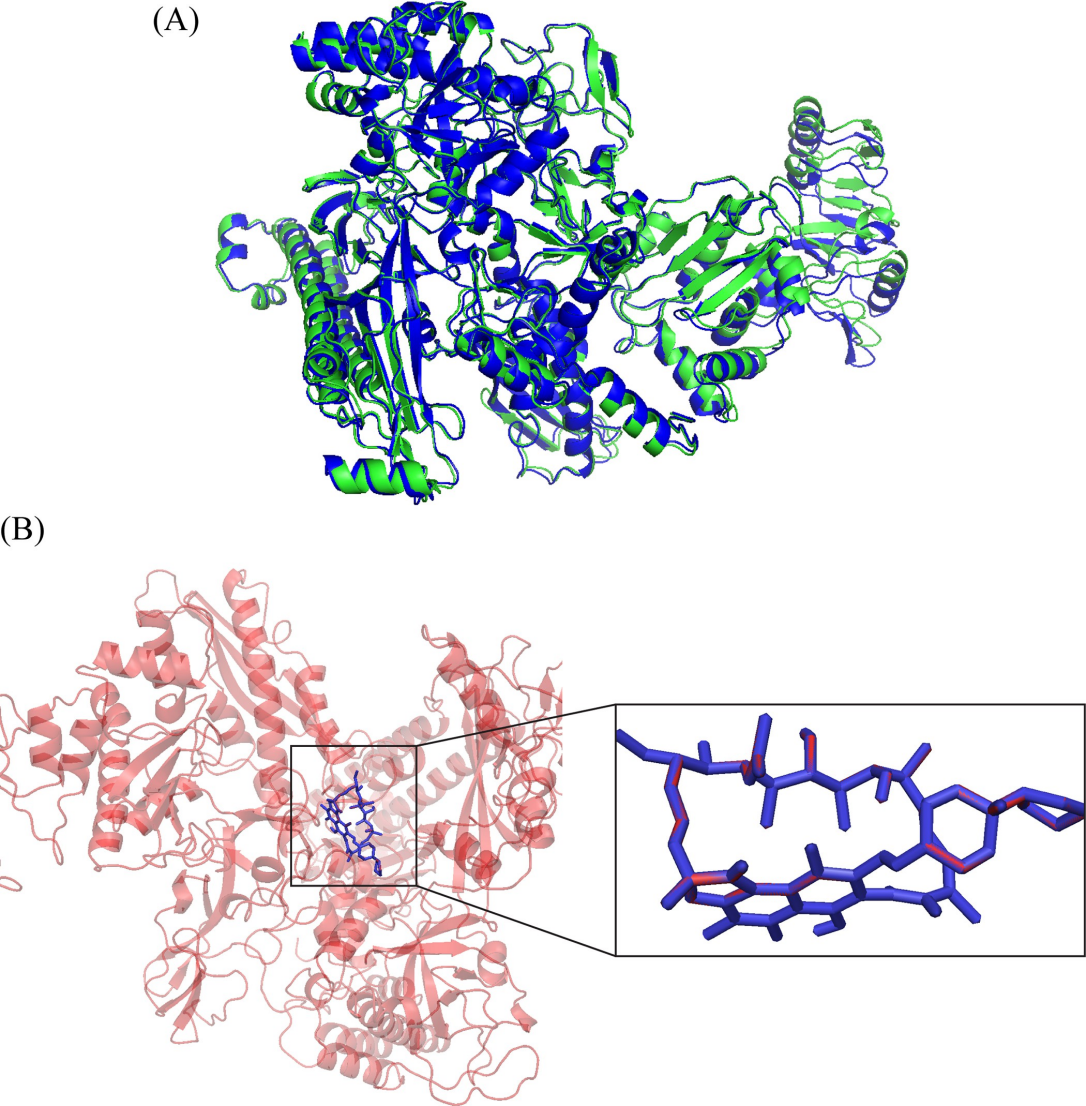
(A) The alignment of apo and holo-states of the RpoB to find the conformational changes, no change was observed as the alignment showed 0.771 RMSD value (Green structure shows the apo state while blue shows the holo states). (B) The representation of native and redocked pose with 0 RMSD value. Red sticks show the native pose while blue shows the redocked pose.

Prior to the docking of the metabolite’s library to the receptors, the positive controls were docked to the wildtype receptor to find the accuracy of the docking tool. In the docking protocol, the receptor was kept rigid, and ligand was flexible. Further, the protein states in the apo and holo form were compared to find the structural changes in the protein in the presence of ligand. The carbon alpha atoms of apo and holo states were aligned to find the deviation between them. The RMSD between two states was 0.77 Å (Fig 2A). Additionally, the control was redocked to the protein to find the accuracy of the tool. After redocking, the native and redocked poses were compared and it was observed that the redocking produced exactly similar results to the native docking 0 RMSD (Fig 2B). After validation, the library of echinoderm’s metabolites was docked against wildtype and mutant Escherichia coli RNA polymerase. The binding poses were analyzed based on the glide score, and the 10 compounds against each protein were selected and compared with the control compounds (Table 1). In wildtype protein docking analysis, the controls showed binding affinities in the range of -5.49 to -4.59, while the selected compounds showed affinities in the range of -9.26 to -8.43 kcal/mol. For the D516V mutant, the docking scores of controls were in the range of -3.78 to -3.02 and the docking scores of selected compounds showed affinities in the range of -8.88 to -8.10 kcal/mol. In the case of H526Y mutant, the hits showed binding affinities ranging from -7.87 to -7.20 which were better than the control binding affinities. Similarly, hits showed better affinities against the S531L mutant than the controls. Additionally, the ADMET properties were calculated by QikProp tool. The ADMET terms QPlogPo/w, QPlogHERG, QPPCaco, QPlogBB, and QPlogKhsa were predicted. QPlogPo/w determines the octanol/water partition coefficient, a value within a range of –2.0 to 6.5 is good. The QPlogHERG descriptor refers to the predicted IC50 value for the blockage of HERG K+ channels, which is an indicator for cardiotoxicity. QPPCaco predicts apparent Caco-2 cell permeability in nanometers per second. QPlogBB represents the predicted brain/blood partition coefficient, which is crucial in assessing a compound’s ability to cross the blood-brain barrier and QPlogKhsa refers to the prediction of binding to human serum albumin, with a recommended range of -1.5 to 1.5 for 95% of known drugs. The analysis revealed that all the compounds have the octanol/water partition coefficient within the acceptable range. Similarly, the first compound in the selected top 10 compounds possess the ability to penetrate the blood-brain barrier and has the ability to block the HERG K+ channel (Table 2). So, the top compound in the ADMET properties was selected for the stability analysis.

**Table 1:** List of compounds virtually screen based on their interactions score.

No.	Compounds	Glide Score	No.	Compounds	Glide Score
RpoB Wildtype (5UAC)			RpoB H526Y mutant (5UAQ)		
**1**	CMNPD2176	-9.266	**1**	CMNPD2177	-7.873
**2**	CMNPD2173	-9.264	**2**	CMNPD4101	-7.809
**3**	CMNPD13821	-8.88	**3**	CMNPD1688	-7.63
**4**	CMNPD4633	-8.779	**4**	CMNPD23099	-7.609
**5**	CMNPD24273	-8.658	**5**	CMNPD23098	-7.564
**6**	CMNPD17483	-8.649	**6**	CMNPD2176	-7.315
**7**	CMNPD18506	-8.624	**7**	CMNPD2520	-7.241
**8**	CMNPD4631	-8.597	**8**	CMNPD25642	-7.223
**9**	CMNPD31497	-8.465	**9**	CMNPD7213	-7.213
**10**	CMNPD7190	-8.435	**10**	CMNPD29869	-7.204
**11**	*Rifampicin	-5.499	**11**	*Rifampicin	-2.620
**12**	*Rifamycin	-5.263	**12**	*Rifamycin	-2.576
**13**	*Rifapentine	-4.594	**13**	*Rifapentine	-1.377
**RpoB D516V mutant (5UAH)**			**RpoB S531L mutant (5UAL)**		
**1**	CMNPD13873	-8.887	**1**	CMNPD11620	-8.307
**2**	CMNPD18509	-8.826	**2**	CMNPD5273	-7.785
**3**	CMNPD2150	-8.725	**3**	CMNPD27050	-7.777
**4**	CMNPD25663	-8.669	**4**	CMNPD3093	-7.772
**5**	CMNPD18501	-8.596	**5**	CMNPD1687	-7.747
**6**	CMNPD21857	-8.58	**6**	CMNPD29869	-7.711
**7**	CMNPD25662	-8.381	**7**	CMNPD27049	-7.655
**8**	CMNPD25665	-8.328	**8**	CMNPD14657	-7.446
**9**	CMNPD4099	-8.277	**9**	CMNPD15630	-7.395
**10**	CMNPD6488	-8.105	**10**	CMNPD9366	-7.339
**11**	*Rifampicin	-6.523	**11**	*Rifampicin	-2.564
**12**	*Rifamycin	-5.486	**12**	*Rifamycin	-2.096
**13**	*Rifapentine	-4.602	**13**	*Rifapentine	-1.207

*Control

**Table 2:** The ADMET properties of the selected compounds calculated by QikProp.

Compounds	QPlogPo/w	QPlogHERG	QPPCaco	QPlogBB	QPlogKhsa
**RpoB Wildtype (5UAC)**					
CMNPD2176	-1.931	-5.865	0.086	-2.844	-0.589
CMNPD2173	-2.505	-5.138	0.025	-8.866	-2.766
CMNPD13821	2.794	-0.543	0.017	-9.142	-2.11
CMNPD4633	-1.883	-2.819	0.009	-8.111	-2.9
CMNPD24273	-1.843	-3.383	0.003	-9.53	-2.82
CMNPD17483	-1.511	-4.95	0.035	-8.345	-2.325
CMNPD18506	-3.085	-2.883	0.003	-9.008	-3.442
CMNPD4631	-2.565	-2.737	0.015	-7.682	-3.255
CMNPD31497	-5.506	-7.128	0.03	-10.516	-3.926
CMNPD7190	7.772	-6.362	13.607	-6.775	0.758
**RpoB D516V mutant (5UAH)**					
CMNPD13873	-0.739	-6.087	-7.972	-2.962	0.006
CMNPD18509	-4.166	-0.663	-5.362	-6.063	0.32
CMNPD2150	-1.829	-4.192	-6.536	-5.117	0.04
CMNPD25663	-3.959	-0.996	-5.696	-6.132	0.336
CMNPD18501	-2.016	-2.705	-7.616	-1.431	0
CMNPD21857	-2.102	-2.254	-7.464	-2.625	0.003
CMNPD25662	-4.474	-0.773	-5.325	-6.231	0.209
CMNPD25665	-3.43	-1.514	-5.779	-6.43	0.579
CMNPD4099	-1.861	-2.617	-6.622	-4.254	0.087
CMNPD6488	-3.697	-0.721	-5.592	-4.073	0.008
**RpoB H526Y mutant (5UAQ)**					
CMNPD2177	-1.922	-5.622	-6.735	-1.828	0.038
CMNPD4101	-2.962	-3.242	-6.376	-5.043	0.005
CMNPD1688	-3.137	-3.404	-6.119	-5.107	0.005
CMNPD23099	-1.427	-4.362	-6.296	-6.729	0.799
CMNPD23098	-0.599	-5.285	-6.701	-6.872	1.115
CMNPD2176	-1.848	-3.117	-6.735	-4.583	0.069
CMNPD2520	1.65	-3.724	-5.478	-4.754	74.506
CMNPD25642	-1.017	-4.51	-5.969	-6.406	2.086
CMNPD7213	-3.117	-1.371	-5.856	-4.267	0.03
CMNPD29869	-1.5	-4.477	-7.514	-3.357	0.002
**RpoB S531L mutant (5UAL)**					
CMNPD11620	-1.731	-5.927	-6.84	-2.38	0.01
CMNPD5273	-5.087	-1.55	-5.312	-5.218	0.004
CMNPD27050	-0.673	-3.67	-7.141	-2.35	0.008
CMNPD3093	-2.106	-3.765	-6.444	-4.934	0.032
CMNPD1687	-3.056	-2.319	-6.119	-4.557	0.005
CMNPD29869	-1.875	-4.607	-7.514	-3.39	0.001
CMNPD27049	-0.728	-3.684	-7.179	-2.348	0.007
CMNPD14657	2.458	-6.143	-5.759	-5.924	44.149
CMNPD15630	2.039	-5.948	-7.466	-3.654	2.046
CMNPD9366	-1.481	-2.48	-4.373	-5.178	1.276

"QPlogHERG (<-5), QPlogPo/w (-2.0 to 6.5), QPlogBB (-3.0 to 1.2), QPPCaco (<25 poor, >500 great), and QPlogKhsa (-1.5 to 1.5)".

### 3.3. Binding pose analysis

The study examined the molecular associations between echinoderm metabolites and protein targets, encompassing RpoB wildtype, RpoB D516V mutant, RpoB H526Y mutant, and RpoB S531L mutant. Based on the glide scores, the compound exhibiting the highest potential for each target was selected and subsequently subjected to further analysis to investigate its molecular interactions and the durability of its binding interactions.

#### 3.3.1. RpoB wildtype

**Fig 3:**
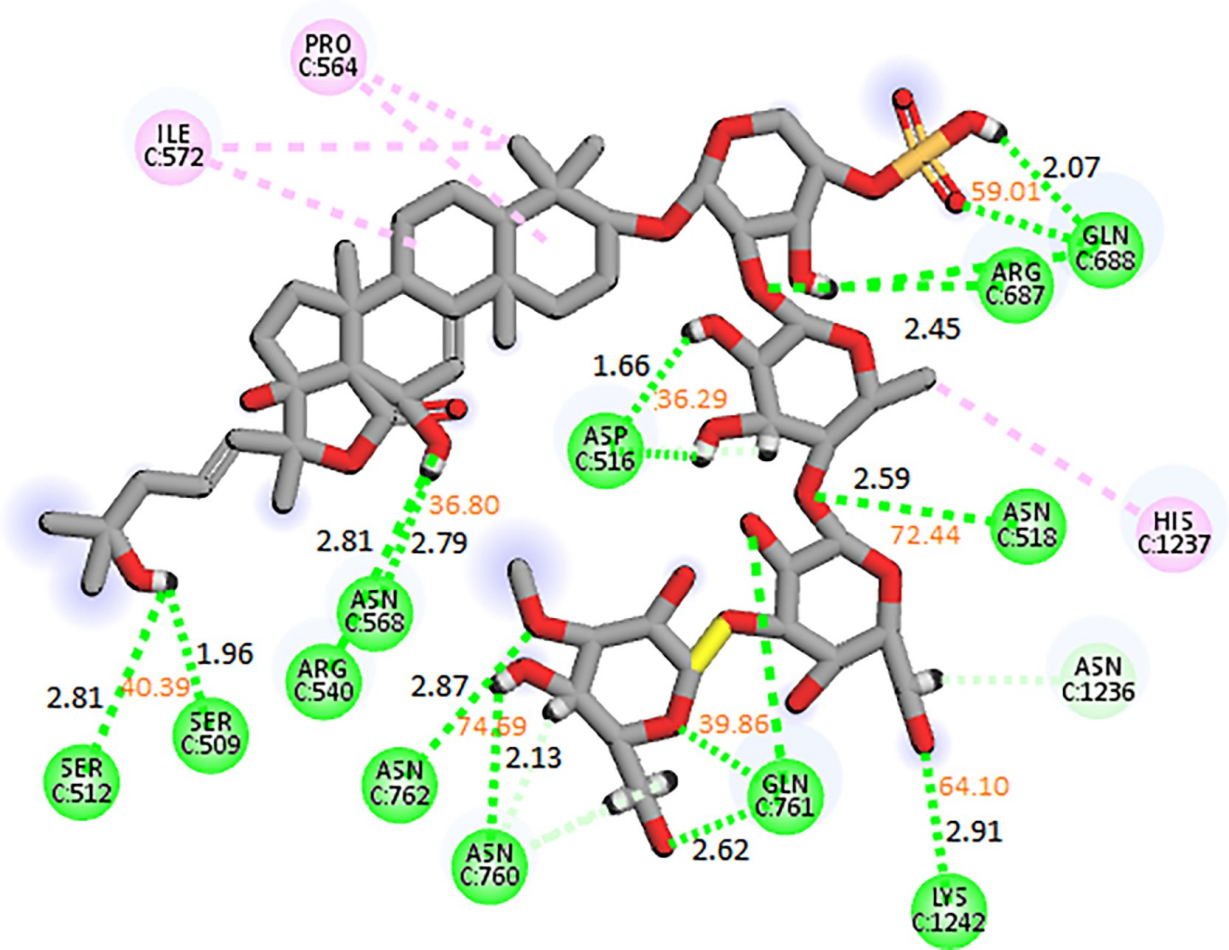
The molecular interactions of CMNPD2176 against wildtype RpoB shown by green sphere (Hydrogen bonds) and magenta spheres (Alkyl interaction/hydrophobic). The distance between the hydrogen bonding residues is indicated by black color while orange values showed the bond angles.

**Fig 4:**
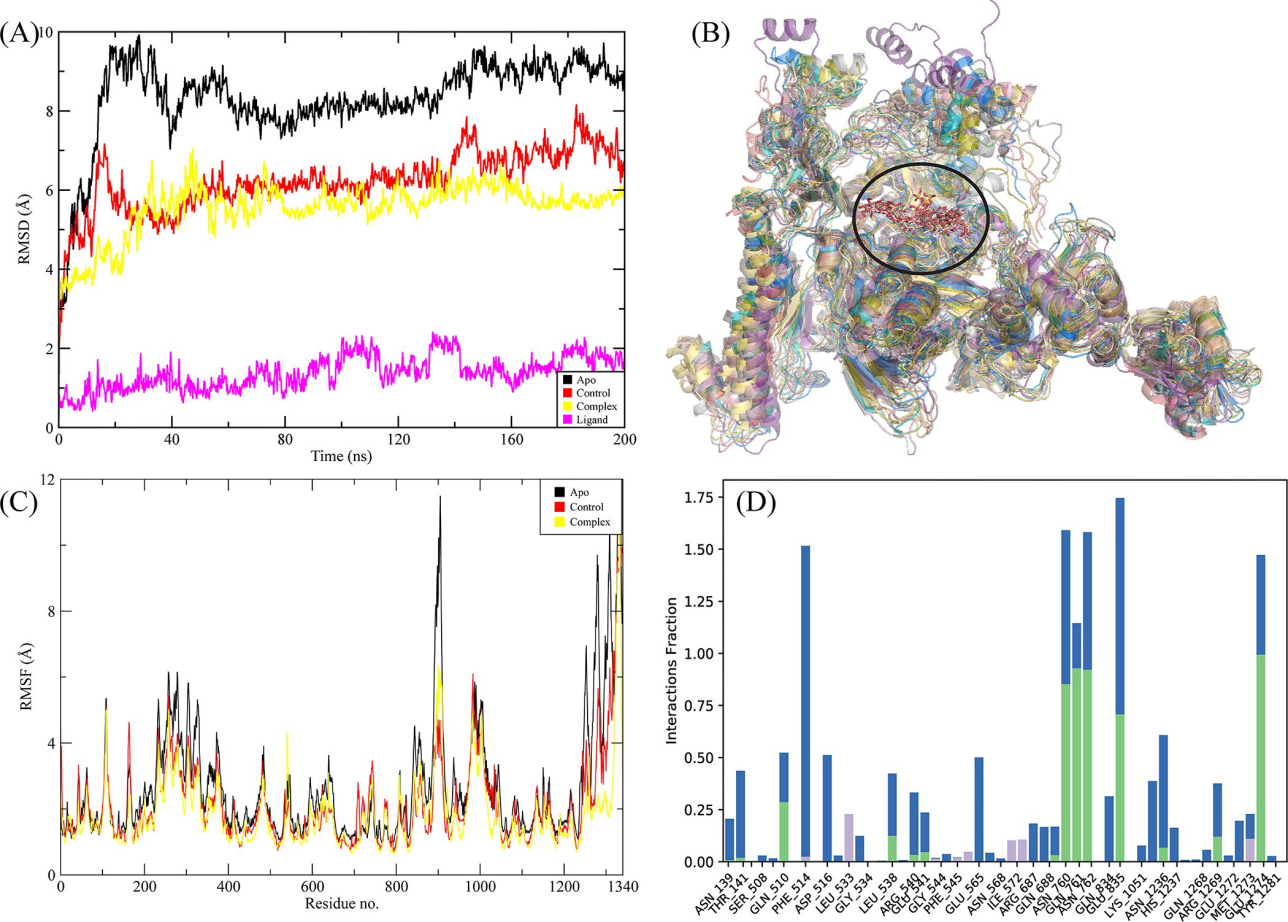
The MD trajectory analysis of the apo and wildtype RpoB complex. (A) RMSD of C-alpha atoms of apo, control, complex and ligand atoms. (B) Aligned snapshots of md trajectory extracted at regular intervals. (C) The RMSF comparison of apo, control and complex. (D) Protein-ligand contacts calculated during simulation.

**Fig 5:**
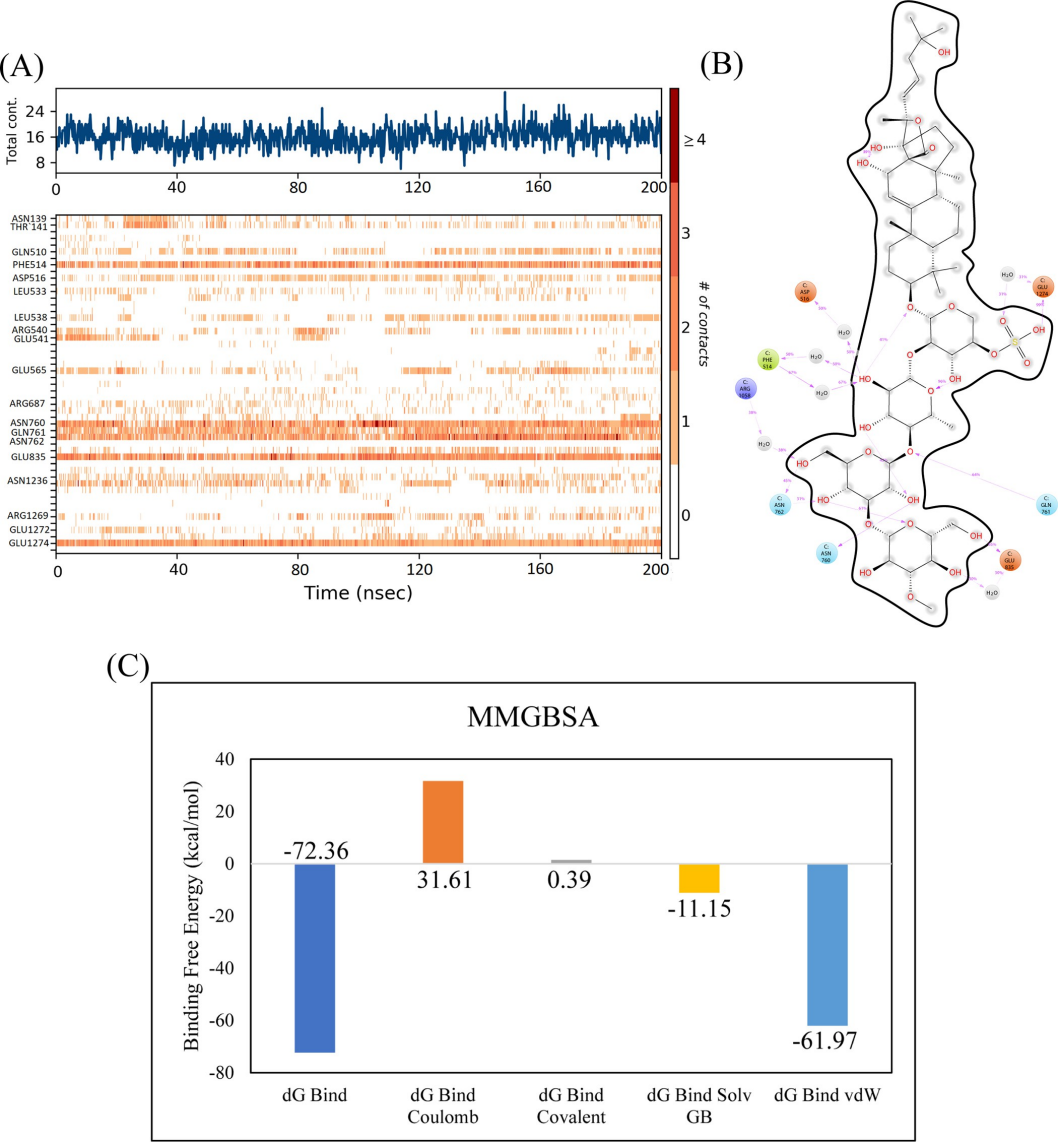
(A) The heat map of the ligand contacts observed during 200 ns simulation (B) Tendency of the interacting residues with the ligand during simulation. (C) The contribution of the energy components in total binding free energy in wildtype RpoB complex.

Of the echinoderm’s metabolites, CMNPD2176 showed the highest binding affinity against wildtype RpoB. The molecular interactions showed that the compound made 12 hydrogen bonds with polar residues Ser512, Ser509, Arg540, Asn568, Asn762, Asn760, Gln761, Asp516, Asn518, Arg687, Gln688, and Lys1242. It also made three hydrophobic interactions with Ile572, Pro564, and His1237. The distance between the hydrogen bonded residues and the bond angles are shown in Fig 3. The stability of the complex was analyzed by conducting a 200 ns simulation and comparing it with the apo RpoB and control complex. The RMSD of C-alpha atoms in the apo protein was in the range of ~8–9 Å throughout the simulation after attaining equilibrium at 20 ns, while the RMSD of the control complex remained in the range of 6–7 Å. In comparison, the RMSD of the hit complex was lower than the apo protein and control with RMSD less than 6 Å throughout the simulation indicating the stability of the complex. Further, the RMSD of ligand was also calculated to find the confirmational changes in the ligand. The RMSD of ligand atoms was lower than 2 Å exhibiting the stable confirmation of ligand during simulation (Fig 4A). To explore the stability of protein-ligand complex, the snapshots from the md trajectory were extracted at 0, 20, 40, 60, 80, 100, 120, 140, 160, 180, 200 ns and aligned. The aligned snapshots revelated that the ligand was remained tightly bound to the binding site (Fig 4B). The structural dynamics of the protein residues were analyzed by calculating the RMSF values, which show the flexibility of protein residues in response to the binding of this ligand during simulation. From the RMSF values, it was observed that most of the residues remained rigid during the simulation except for the residues ranging from 890 to 910 which showed more fluctuations in apo protein as compared to the complex (Fig 4C). We observed that the primary connections between the protein and ligands were ionic bonds, hydrogen bonds, and hydrophobic interactions. These interactions play a vital role in maintaining the stability of the protein-ligand complex and influencing its functional traits. The residues involved in hydrogen bonding were Gln510, Leu538, Arg540, Glu541, Gln688, Asn760, Gln761, Asn762, Asn1236, Arg1269, and Glu1274. There was no residue involved in the ionic interactions (Fig 4D). The interacting residues were further explored by the heat map of ligand contacts during 200 ns simulation. The interacting residues made orange band showing the number of contacts during simulation. The darker the color, more the contacts between ligand and residues. The heat map showed that Phe514, Asn760, Gln761, Asn762, Glu835, and Glu1274 formed more than 1 contacts with ligand throughout the simulation (Fig 5A). Among these interacting residues, Glu1274 exhibited the highest tendency for binding, with interactions observed during 99% of the total frames (Fig 5B). Furthermore, the binding free energy of the complex was calculated by the prime-MMGBSA module. The binding free energy was the total of Van der Waals, Coulomb, Solvation, and Covalent energies. The van der Waals energy contribution was -61.97, solvation was -11.15, covalent energy was 0.39, coulombic energy was 31.61, and the total binding free energy of the complex was -72.36 kcal/mol as shown in Fig 5C.

#### 3.3.2. RpoB D516V mutant

**Fig 6:**
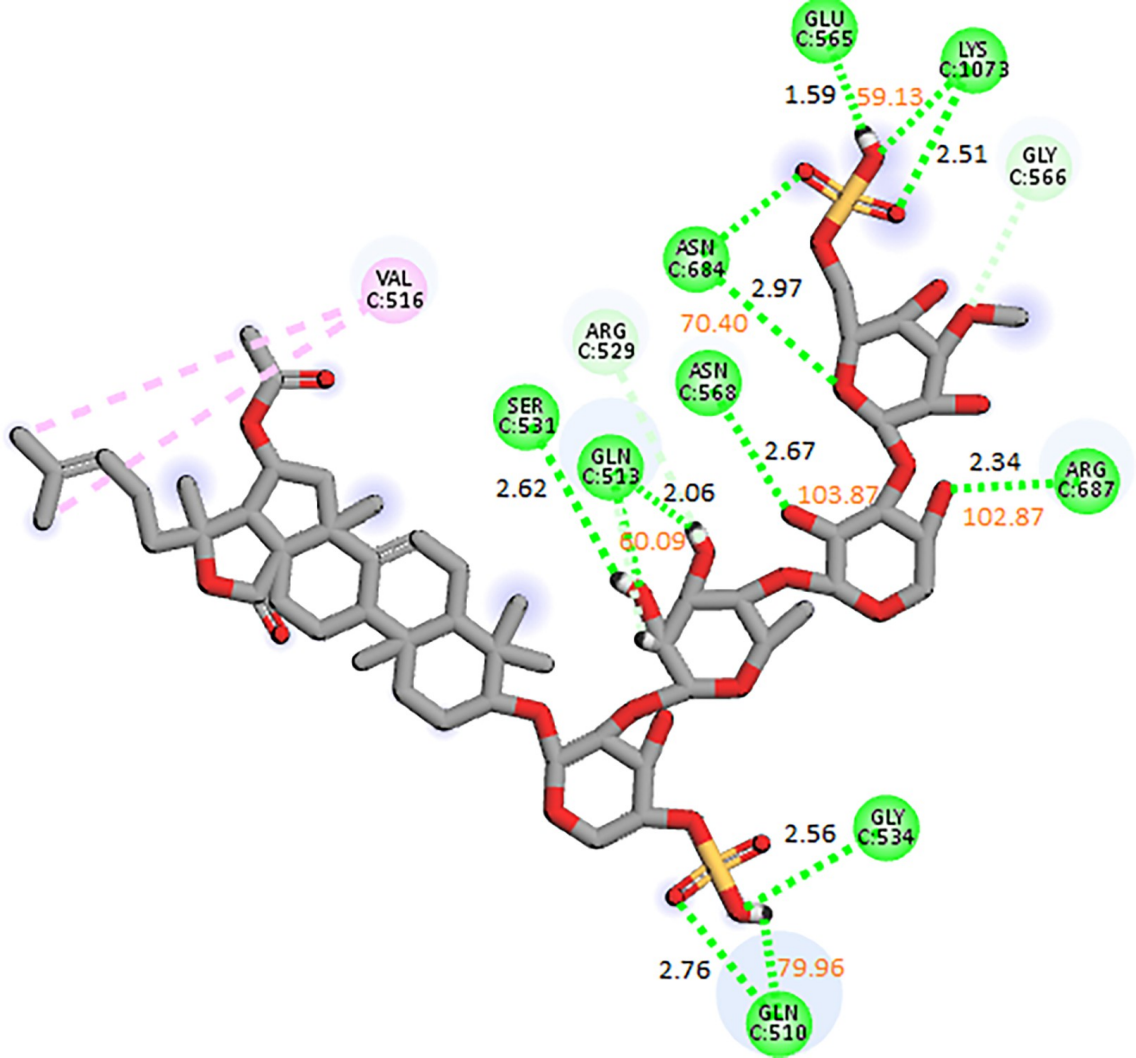
The molecular interactions of CMNPD13873 against RpoB D516V mutant. The distance between the hydrogen bonding residues is indicated by black color while orange values showed the bond angles.

**Fig 7:**
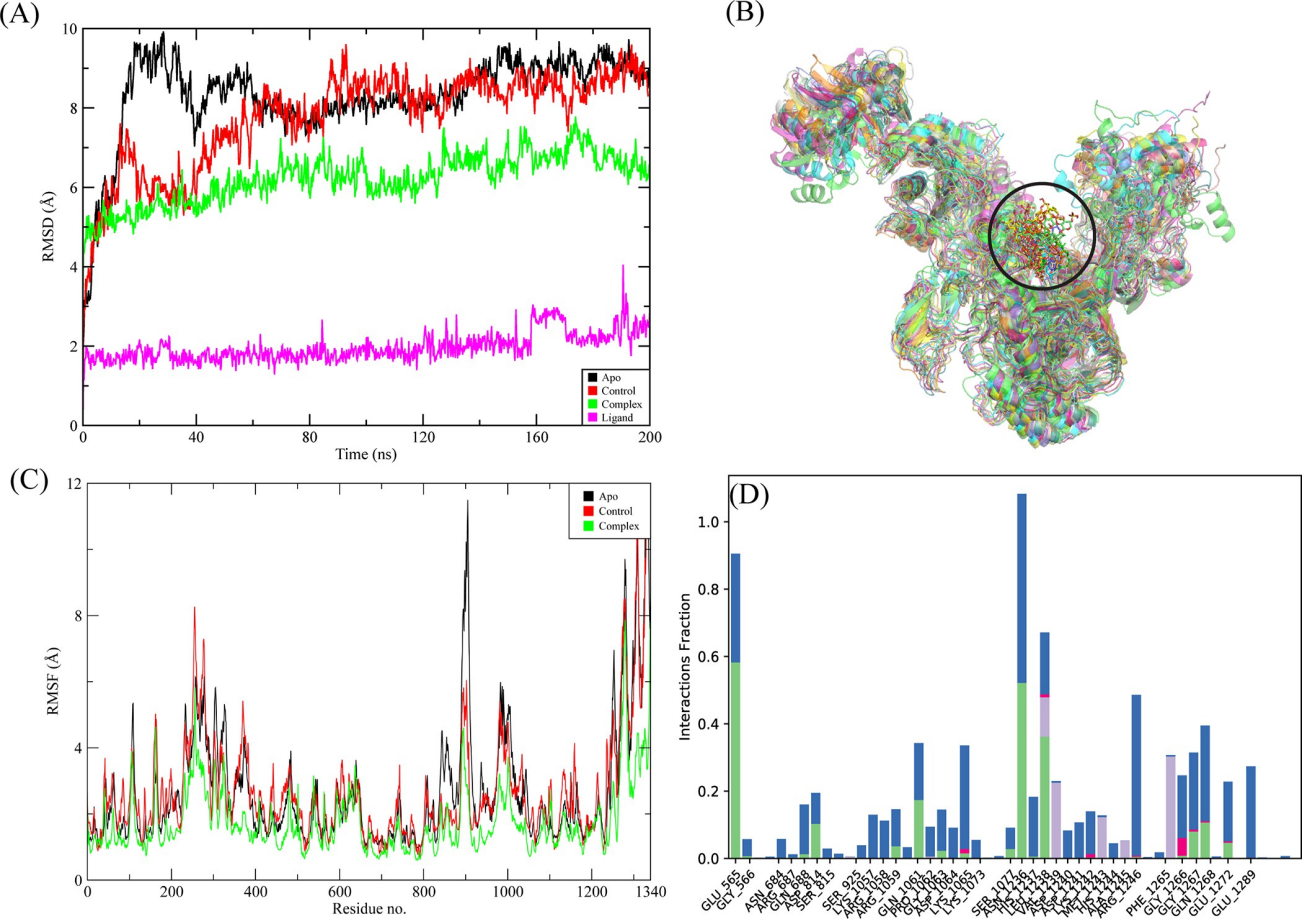
The MD trajectory analysis of the RpoB D516V mutant complex. (A) RMSD of C-alpha atoms of apo, control, complex and ligand atoms. (B) Aligned snapshots of md trajectory extracted at regular intervals. (C) The RMSF comparison of apo, control and complex. (D) Protein-ligand contacts calculated during simulation.

**Fig 8:**
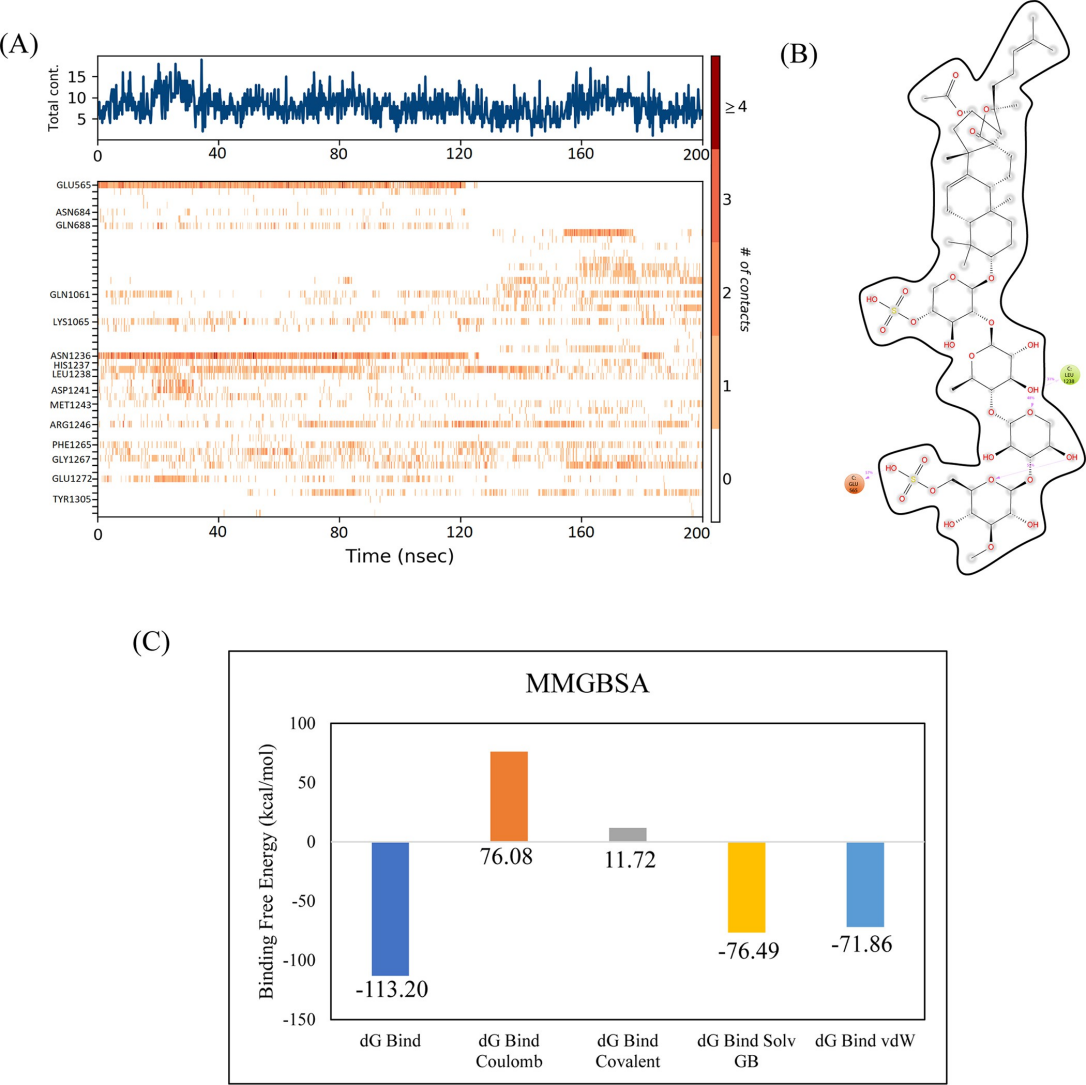
(A) The heat map of the ligand contacts observed during 200 ns simulation (B) Tendency of the interacting residues with the ligand during simulation. (C) The contribution of the energy components in total binding free energy in wildtype RpoB D516V mutant complex.

The docking analysis of the RpoB D516V mutant revealed that CMNPD13873 showed the highest binding affinity among the selected ligands, so it was selected for the molecular interaction and stability analysis. The molecular interactions showed that CMNPD13873 made hydrogen bonds with nine residues: Gln510, Gly534, Arg687, Ser531, Gln513, Asn568, Asn684, Glu565, and Lys1073. It also made one alkyl interaction with Val516 as shown in Fig 6. The RMSD of the C-alpha atoms in the control maintained the same range as the apo protein, i.e., ~ 8–9 Å, after attaining equilibrium at 50 ns, while the RMSD of the complex was in the range of 6–7 Å lower than the apo and control. Similarly, the RMSD of ligand atoms was less than 2 Å till 160 ns and then increased to 3 Å towards the end of simulation (Fig 7A). The alignment of the snapshots revealed that the ligand remained tightly bound to the binding pocket and did not show confirmational changes during the simulation (Fig 7B). The RMSF analysis revealed that the complex and control protein showed the same trend, i.e., most of the residues remained rigid except the residues from 890 to 910, where apo protein showed more fluctuations (Fig 7C). In protein-ligand contact analysis, the specific residues involved in hydrogen bonding were Glu565, Asp814, Arg1059, Gln1061, Asn1236, Leu1238, Gly1267, and Glu1272 (Fig 7D). The ligand heat map indicated that Glu565, Asn1236, and Leu1238 made strong contact throughout the simulation (Fig 8A). Among these interacting residues, Glu565 exhibited the highest tendency for binding, with interactions observed during 57% of the total frames (Fig 8B). The binding free energy calculation showed that the total binding free energy of the complex was -113.20 kcal/mol. The other energy components are shown in Fig 8C.

#### 3.3.3. RpoB H526Y mutant

**Fig 9:**
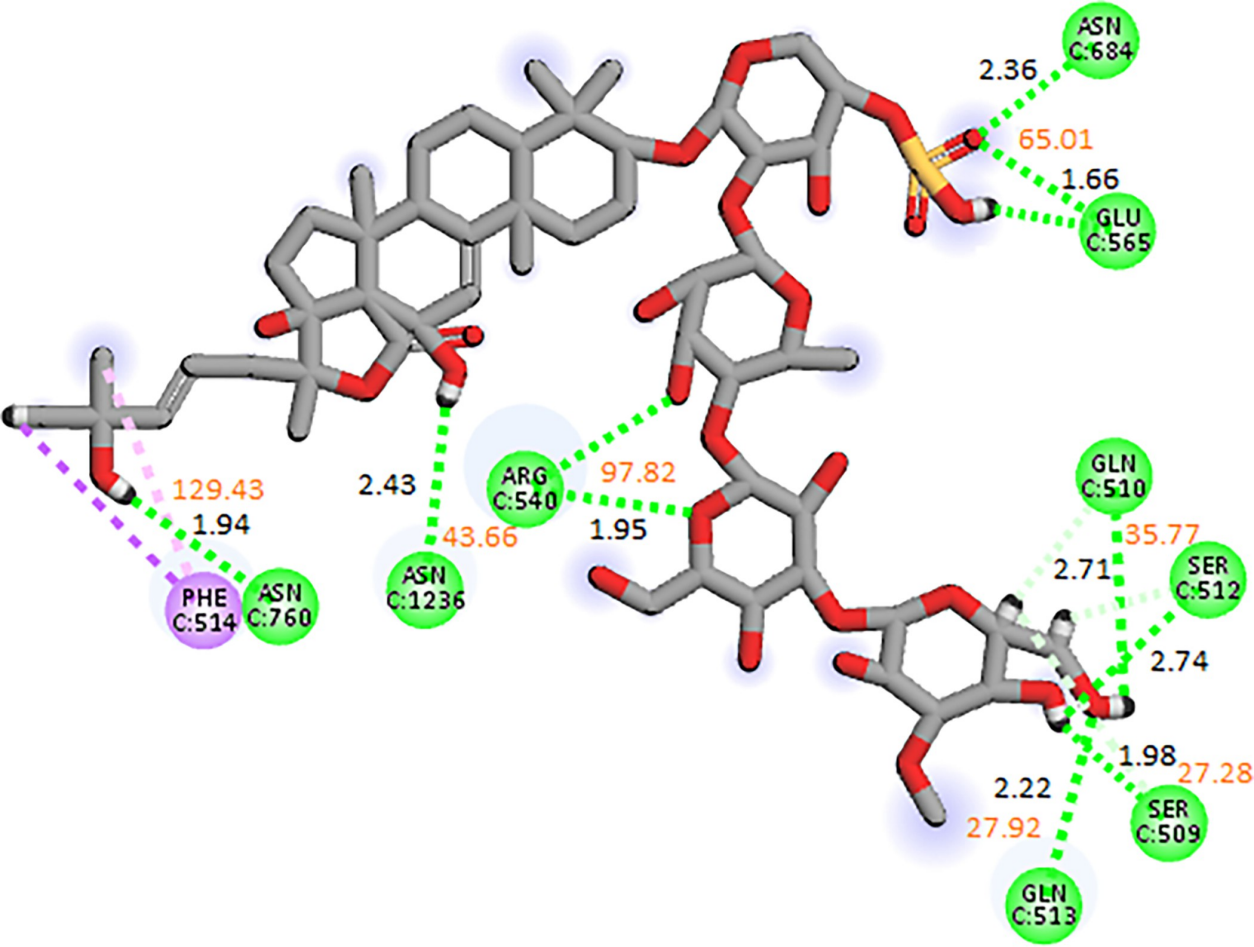
The molecular interactions of CMNPD2177 against RpoB H526Y mutant.

**Fig 10:**
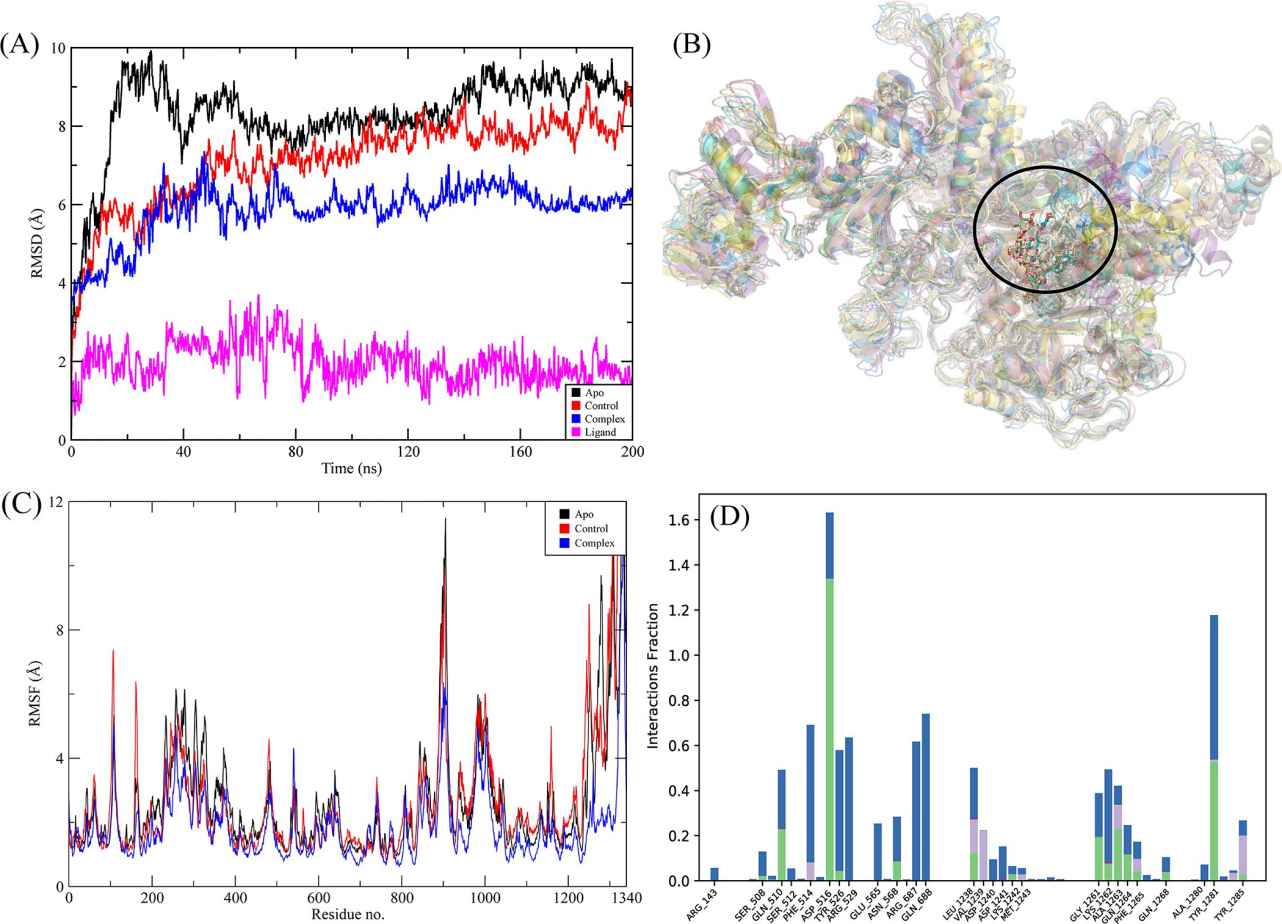
The MD trajectory analysis of the RpoB H526Y mutant complex. (A) RMSD of C-alpha atoms of apo, control, complex and ligand atoms. (B) Aligned snapshots of md trajectory extracted at regular intervals. (C) The RMSF comparison of apo, control and complex. (D) Protein-ligand contacts calculated during simulation.

**Fig 11:**
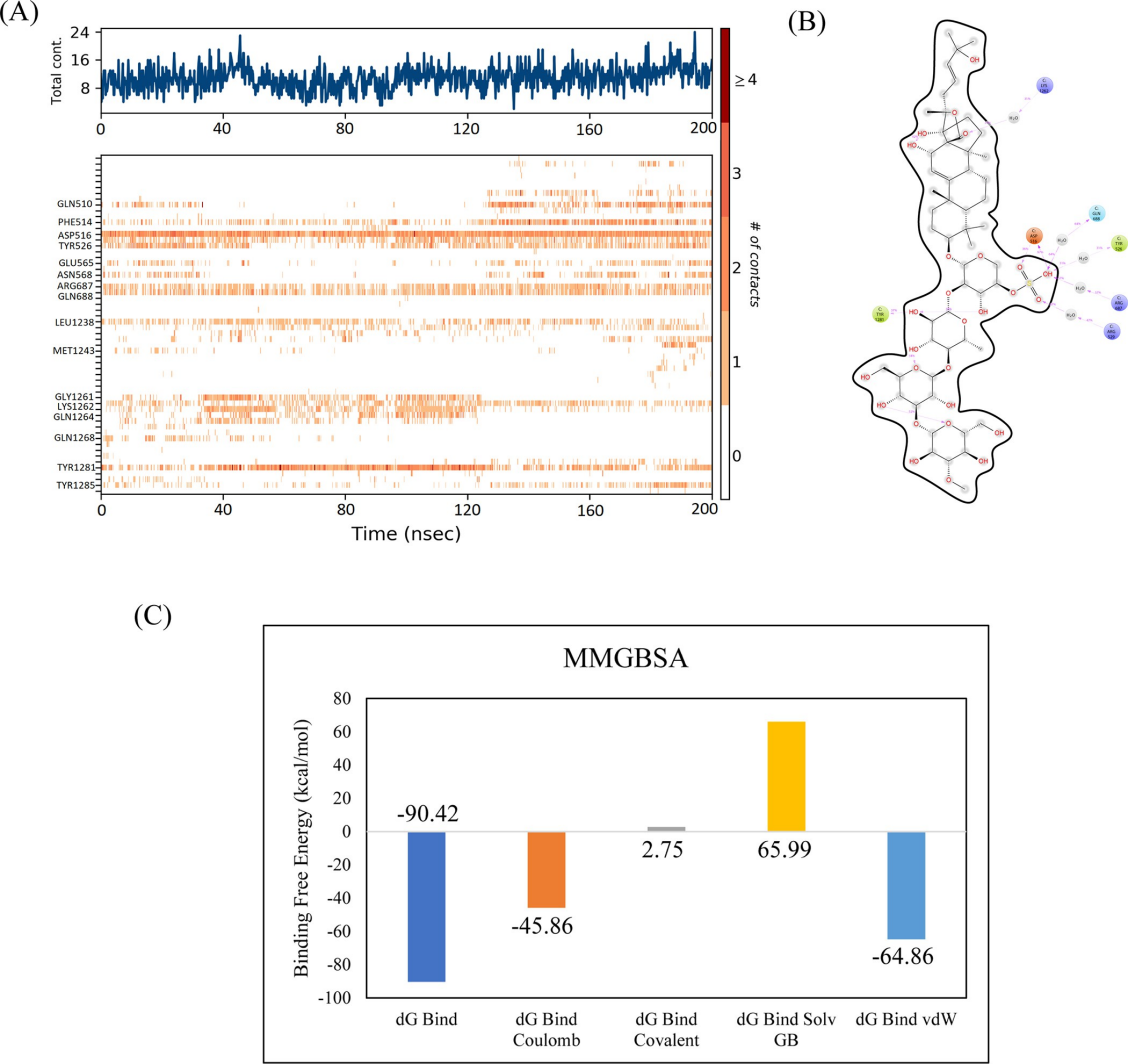
(A) The heat map of the ligand contacts observed during 200 ns simulation (B) Tendency of the interacting residues with the ligand during simulation. (C) The contribution of the energy components in total binding free energy in RpoB H526Y mutant complex.

CMNPD2177 showed the highest binding affinity against the RpoB H526Y mutant. The molecular interactions showed that CMNPD2177 made nine hydrogen bonds with Gln513, Ser509, Ser512, Gln510, Glu565, Asn684, Arg540, Asn760, and Asn1236 and one pi-sigma interaction with Phe514, as shown in Fig 9. The RMSD of the C-alpha atoms in the control was in the range of 8 Å less than apo protein, while the RMSD of complex was in the range of 6 Å lower than both apo and control. Similarly, the RMSD of ligand atoms was less than 2 Å throughout the simulation except for an interval of 40 to 80 ns (Fig 10A). The alignment of the snapshots revealed that the ligand remained tightly bound to the binding pocket and did not show confirmational changes during the simulation (Fig 10B). The RMSF analysis showed that the residues remained rigid during the simulation except for the residues from 890 to 910, both in the apo protein and complex (Fig 10C). In protein-ligand contact analysis, the residues involved in hydrogen bonding were Ser508, Gln510, Asp516, Tyr526, Asn568, Leu1238, Lys1242, Gly1261, Lys1262, Ala1263, and Tyr1281 (Fig 10D). The ligand heat map indicated that Phe514, Tyr526, Arg687, Leu1238, Gly1261, and Tyr1281 made strong contact throughout the simulation (Fig 11A). Among these interacting residues, Asp516 exhibited the highest tendency for binding, with interactions observed during 97% of the total frames (Fig 11B). The binding free energy calculation showed that the total binding free energy of the complex was -90.42 kcal/mol. The other energy components are shown in Fig 11C.

#### 3.3.4. RpoB S531L mutant

**Fig 12:**
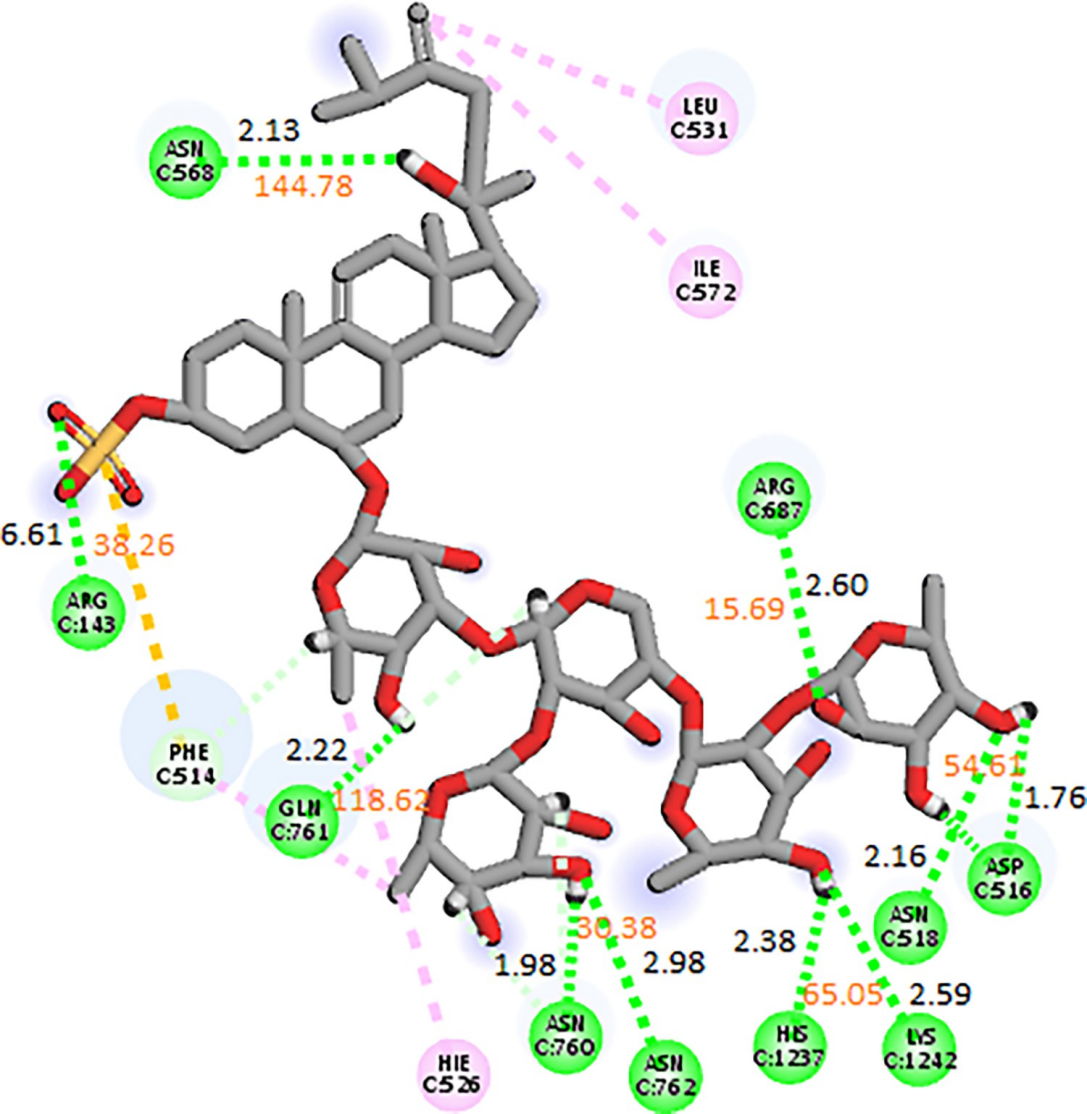
The molecular interactions of CMNPD11620 against RpoB S531L mutant.

**Fig 13:**
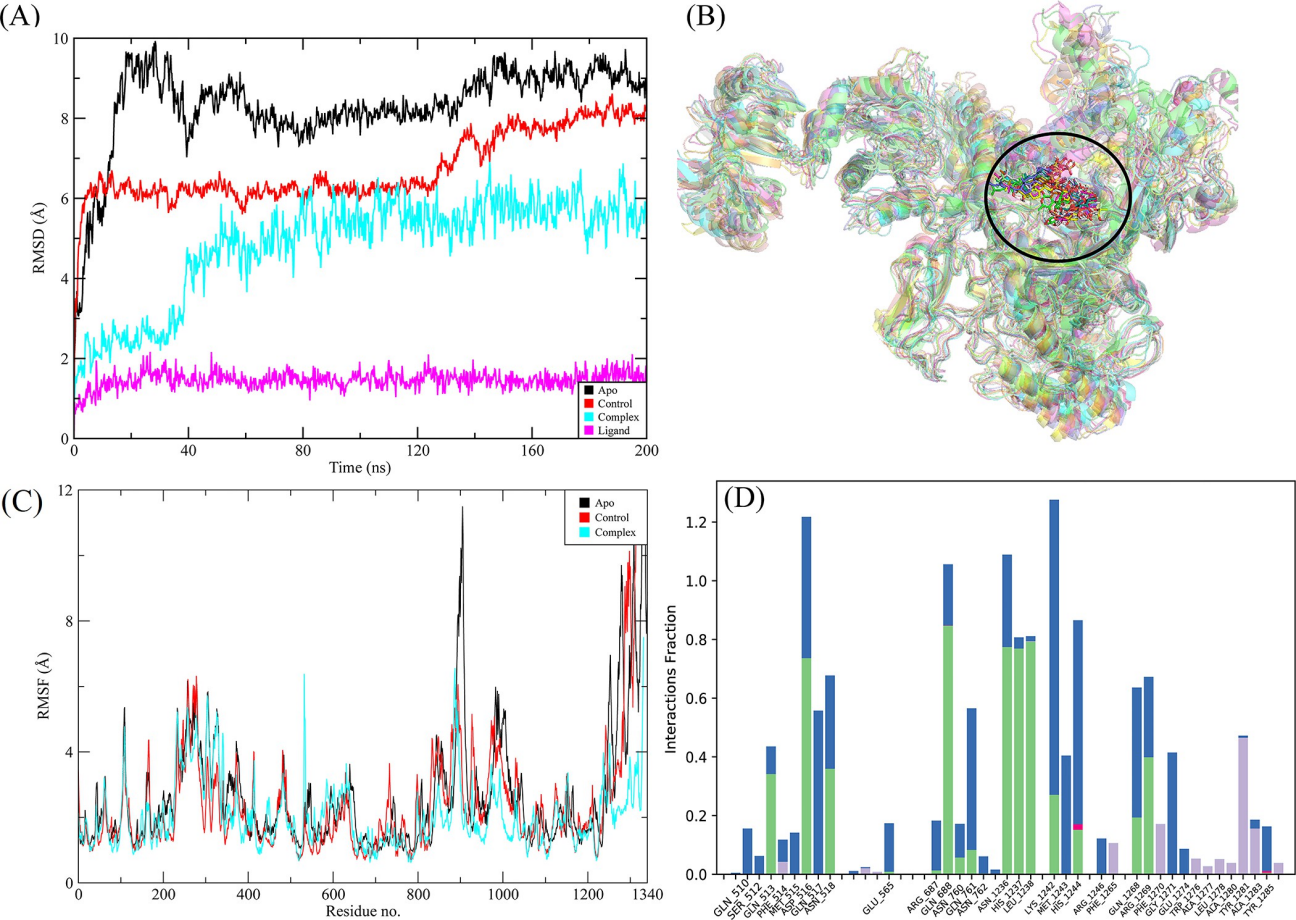
The MD trajectory analysis of the RpoB S531L mutant complex. (A) RMSD of C-alpha atoms of apo, control, complex and ligand atoms. (B) Aligned snapshots of md trajectory extracted at regular intervals. (C) The RMSF comparison of apo, control and complex. (D) Protein-ligand contacts calculated during simulation.

**Fig 14:**
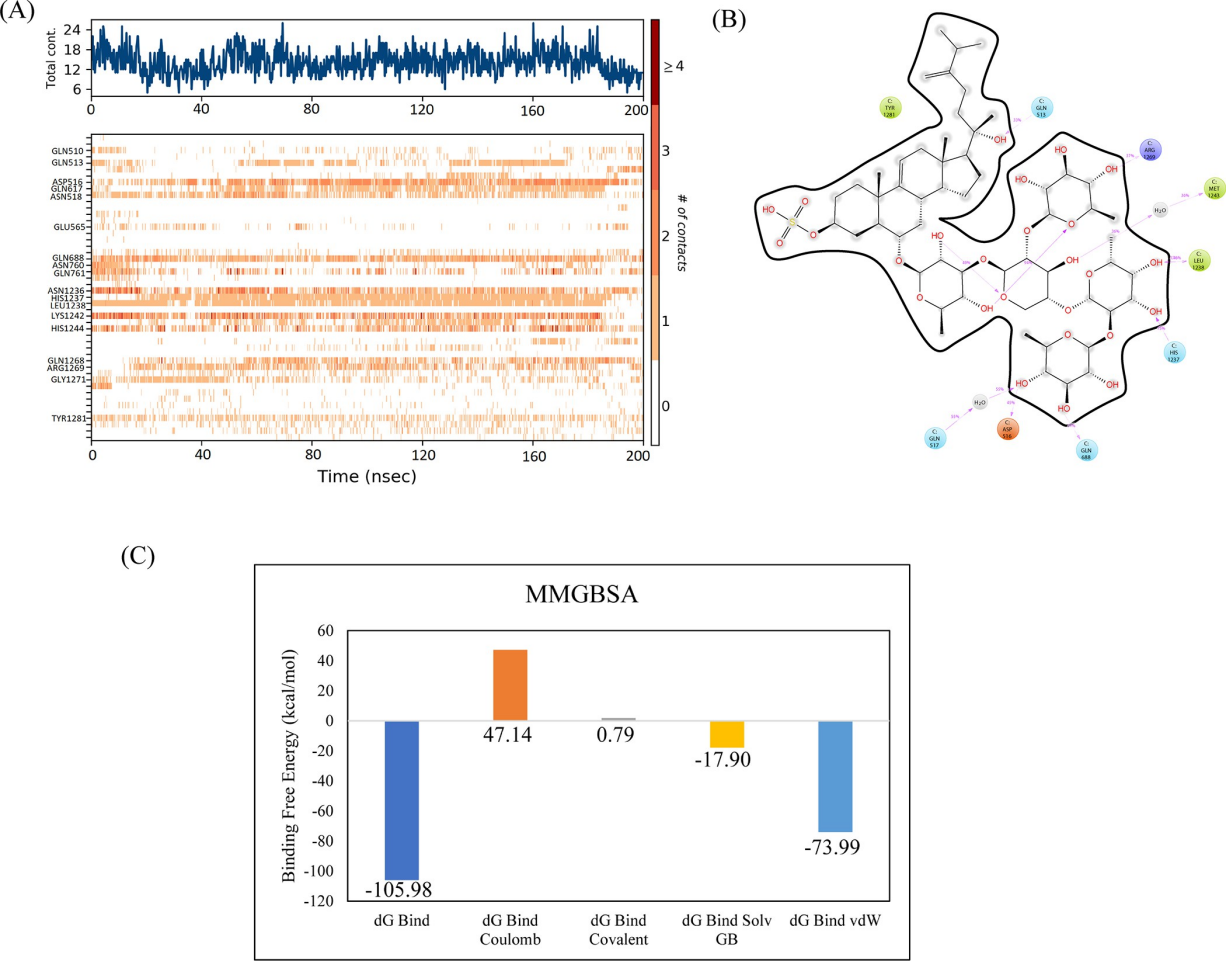
(A) The heat map of the ligand contacts observed during 200 ns simulation (B) Tendency of the interacting residues with the ligand during simulation. (C)The contribution of the energy components in total binding free energy in RpoB S531L mutant complex.

In the docking studies of the RpoB S531L mutant, CMNPD11620 showed the highest binding affinity among the selected ligands. The molecular interactions showed that CMNPD11620 made ten hydrogen bonds with Arg143, Asn568, Gln761, Asn760, Asn762, Arg687, Asp516, Asn518, His1237, and Lys1242. It was involved in alkyl interactions with Leu531, Ile572, and His526 as shown in Fig 12. The RMSD of the C-alpha atoms in the control was in the range of 6 Å till 120 ns and then gradually increased to 8 Å towards the end of simulation while the RMSD of complex was less than 6 Å showing more stability. Similarly, the RMSD of ligand atoms was less than 2 Å throughout the simulation (Fig 13A). The alignment of the snapshots revealed that the ligand remained tightly bound to the binding pocket and did not show confirmational changes during the simulation (Fig 13B). The RMSF analysis revealed that the complex and apo protein showed the same trend, i.e., most of the residues remained rigid except the residues from 890 to 910, where apo protein showed more fluctuations (Fig 13C). In protein-ligand contact analysis, the residues involved in hydrogen bonding were Gln513, Asp516, Asn518, Gln688, Asn760, Gln761, Asn1236, His1237, Leu1238, Lys1242, His1244, Gln1268, and Arg1269 (Fig 13D). The ligand heat map indicated that Asp516, Asn518, Gln688, Asn1236, Leu1238, Lys1242, His1244, and Arg1269 made strong contact throughout the simulation (Fig 14A). Among these interacting residues, His1237 exhibited the highest tendency for binding, with interactions observed during 75% of the total frames (Fig 14B). The binding free energy calculation showed that the total binding free energy of the complex was -105.98 kcal/mol. The other energy components are shown in Fig 14C.

## 4. Discussion

Developing an effective drug that targets both wild-type and mutant RpoB in *M*. *tuberculosis* is critical for comprehensive TB management. Wild-type RpoB represents the standard, non-mutated form found in drug-sensitive *M*. *tuberculosis* strains, necessitating adequate therapy for normal TB cases. Simultaneously, mutant RpoB is related to drug-resistant *M*. *tuberculosis* strains, necessitating drugs that may oppose these mutations to effectively battle drug-resistant TB. We ensure comprehensive therapy choices for the varied spectrum of TB cases, from drug-sensitive to drug-resistant forms, by targeting both wild-type and mutant RpoB. This strategy is critical in reducing treatment failure, preventing resistance amplification, optimizing treatment regimens, lowering treatment durations, improving patient adherence, and eventually reducing the worldwide TB burden, particularly in places where drug resistance is an increasing problem. It emphasizes the need for a comprehensive plan to address the multifaceted challenges faced by tuberculosis, ensuring that effective therapies are available to all TB patients, independent of the characteristics of the infecting *M*. *tuberculosis* strains [3, 32–35].

Traditional drug development procedures are expensive and time-consuming, but technological advancement has overcome these constraints. Pharmacokinetics (PK) is critical to accelerating and optimizing the drug development process. Pharmacokinetic studies allow researchers to make informed decisions at various stages of drug development by understanding how drugs are absorbed, distributed, metabolized, and excreted in the body, resulting in increased efficiency and cost savings [36, 37].

The goal of this study is to screen echinoderm metabolites that can inhibit (WT) and mutant RpoB in TB. A library of echinoderm metabolites encompassing 1600 chemicals retrieved from The Comprehensive Marine Natural Products Database was docked to wildtype and mutant *Escherichia coli* RNA polymerase. Molecular docking can be used to screen large compound libraries for potential drug candidates with high binding affinity and specificity to the target protein, thereby speeding up the early stages of drug discovery. The results of molecular docking offer important information about how the ligand and target protein interact, which helps with the logical design and improvement of lead compounds for therapeutic development [38–40]. The docking findings were evaluated using the glide gscore, and the top ten molecules docked against each receptor were chosen. Three control compounds, Rifampicin, Rifamycin, and Rifapentine, were also docked to the receptors. In wild-type protein docking studies, the controls exhibited binding affinities ranging from -5.49 to -4.59 kcal/mol, while the chosen compounds had affinities ranging from -9.26 to -8.43 kcal/mol. The docking scores of controls for the D516V mutant were in the range of -3.78 to -3.02, and the docking scores of selected compounds exhibited affinities in the range of -8.88 to -8.10 kcal/mol. In the case of the H526Y mutant, the binding affinities ranged from -7.87 to -7.20, which were higher than the binding affinities of the controls. Similarly, hits demonstrated higher affinity for the S531L mutant than controls.

Additionally, the physicochemical and ADMET properties of the selected drugs were analyzed. All the compounds were within the allowed ranges for the ADMET characteristics. The molecular weight of a compound indicates its easy distribution in the cells, so the compounds with less weight can easily distribute in the body as compared to compounds with a higher weight. In this regard, a criterion of 500 g/mol was set, and all the molecular weights of all selected compounds fall within this range. QPlogPo/w determines the octanol/water partition coefficient, a value within a range of –2.0 to 6.5 is good. The values of selected hits fall within this range. Assessing ADMET properties early in the drug discovery process allows researchers to identify potential liabilities and optimize compound structures to improve drug-like properties. This test offers information on a candidate’s subcellular localization, ability to cross the blood-brain barrier, intestinal absorption, metabolism, and, most importantly, its potential to affect the body. Overall, ADMET analysis makes it easier to select lead compounds with better pharmacokinetic profiles and lower toxicity, which speeds up drug development and increases the likelihood of clinical success [41–43].

The binding poses of compounds exhibiting the highest binding affinity against the target proteins were scrutinized to elucidate their molecular interactions. Notably, among the selected compounds, CMNPD2176 demonstrated the highest binding affinity against wild-type RpoB, CMNPD13873 exhibited the highest binding affinity against the RpoB D516V mutant, CMNPD2177 displayed the highest binding affinity against the RpoB H526Y mutant, and CMNPD11620 showcased the highest binding affinity against the RpoB S531L mutant. These findings provide insights into the specific molecular interactions underlying the strong binding affinities of these compounds with their respective target proteins.

Molecular Dynamics (MD) simulations and MMGBSA analysis of docked complexes play a crucial role in providing valuable insights into the dynamics, stability, and binding affinity of protein-ligand interactions. These analyses contribute to refining docking predictions, ranking ligand candidates, guiding optimization efforts, and enhancing our comprehension of the molecular mechanisms governing protein-ligand binding. The results from MMGBSA experiments and MD simulations indicate that the investigated compounds remained stable as effective inhibitors within the protein binding pocket [44].

Consequently, the novel drug targets identified in this study hold significant potential for the drug therapeutic industry, offering opportunities to identify inhibitors and develop new drug formulations for tuberculosis control. The combination of molecular docking, MD simulations, and ADMET analysis provides a comprehensive understanding of structure-activity relationships, guiding rational lead optimization strategies for tuberculosis drug discovery. Future efforts, combining computational approaches with experimental validation, have the potential to identify and advance optimized lead compounds with improved efficacy, pharmacokinetics, and safety profiles toward clinical development for tuberculosis treatment.

## 5. Conclusion

This study highlights the potential of echinoderm metabolites as effective inhibitors of both wild-type and mutant forms of *Escherichia coli* RNA polymerase (RpoB) for tuberculosis treatment. Using molecular docking, molecular dynamics simulations, and ADMET analysis, we discovered a number of lead compounds with favorable binding affinity, stability, and pharmacokinetic properties. The stability of the docked hits with the protein was evaluated by MD simulation, and conformational changes were detected by RMSD and RMSF, which indicated that the hits remained stably bound to the protein pocket. Future research should concentrate on further characterizing lead compounds using in vitro and in vivo studies to validate their efficacy and safety profiles. Finally, the development of echinoderm derived RpoB inhibitors holds great promise for meeting the urgent need for new and effective TB treatments, especially in the face of rising drug resistance.
